# Immunochemotherapy with interleukin-2, interferon- **α** and 5-fluorouracil for progressive metastatic renal cell carcinoma: a multicenter phase II study

**DOI:** 10.1054/bjoc.1999.0997

**Published:** 2000-02

**Authors:** C M L van Herpen, R L H Jansen, W H J Kruit, K Hoekman, G Groenewegen, S Osanto, P H M De Mulder

**Affiliations:** Department of Medical Oncology, University Hospital Nijmegen, PO Box 9101, Nijmegen, HB, 6500, The Netherlands; Department of Internal Medicine, University Hospital Maastricht, The Netherlands; Department of Medical Oncology, Daniel den Hoed Clinic/University Hospital, Rotterdam, The Netherlands; Department of Medical Oncology, Free University Amsterdam, The Netherlands; Department of Internal Medicine, University Hospital Utrecht, The Netherlands; Department of Clinical Oncology, University Hospital Leiden, The Netherlands

**Keywords:** immunochemotherapy, renal cell carcinoma, phase II, interleukin-2, interferon-α, 5-FU

## Abstract

In patients with metastatic renal cell carcinoma response rates of 7–26% have been achieved with immunotherapy. A high response rate of 48% in 35 patients has been reported for treatment with the combination of interferon-α (IFN-α), interleukin-2 (IL-2) and 5-fluorouracil (5-FU) (Atzpodien et al (1993 *a*) *Eur J Cancer***29A**: S6–8). We conducted a multicentre phase II study to confirm these results. Metastatic renal cell carcinoma patients were treated as outpatients with an 8-week treatment cycle. Recombinant human IL-2 20 MU m^−2^was administered subcutaneously (s.c.) three times a week (t.i.w) in weeks 1 and 4 and 5 MU m^−2^t.i.w. in weeks 2 and 3. Recombinant human IFN-α 2a 6 MU m^−2^was administered s.c. once in weeks 1 and 4 and t.i.w. in weeks 2 and 3, and 9 MU m^−2^t.i.w. in weeks 5–8. 5-FU (750 mg m^−2^) was given as a bolus injection intravenous once a week in weeks 5–8. The treatment cycle was repeated once in case of response or minor response. Fifty-two patients entered the study. All had undergone a nephrectomy and had progressive metastatic disease. The median WHO-performance status was 1, the median number of metastatic sites was 2 (range 1–5) and the median time between the diagnosis of the primary tumour and the start of treatment was 12.9 months (range 1–153). Among the 51 patients, including four patients with early progressive disease, who were evaluable for response, the response rate was 11.8% (95% confidence interval (CI) 2.9–20.7%), with no complete responses. Median duration of response was 8.3 (range 3.8–22.4+) months. Median survival was 16.5 (range 1.8–30.5+) months. Grade 3/4 toxicity (WHO) occurred in 29/52 (55.8%) of the patients in cycle 1 and in 6/16 (37.5%) of the patients in cycle 2. It consisted mainly of anorexia, fatigue, nausea, fever and leucocytopenia. We cannot confirm the high response rate in patients with metastatic renal cell carcinoma treated with the combination of IFN-α, IL-2 and 5-FU, as described by Atzpodien et al. © 2000 Cancer Research Campaign


*British Journal of Cancer* (2000) **82**(4), 772–776
© 2000 Cancer Research Campaign
DOI: 10.1054/ bjoc.1999.0997, available online at http://www.idealibrary.com on IDEAL®

### Immunochemotherapy with interleukin-2, interferon-α and 5-fluorouracil for progressive metastatic renal cell carcinoma: a multicenter phase II study

**CML van Herpen[1], RLH Jansen[2], WHJ Kruit[3], K Hoekman[4], G Groenewegen[5], S Osanto[6] and PHM De Mulder[1] on behalf of the Dutch Immunotherapy Working Party**

[1]Department of Medical Oncology, University Hospital Nijmegen, PO Box 9101, 6500 HB Nijmegen, The Netherlands; [2]Department of Internal Medicine, University Hospital Maastricht, The Netherlands; [3]Department of Medical Oncology, Daniel den Hoed Clinic/University Hospital, Rotterdam, The Netherlands; [4]Department of Medical Oncology, Free University Amsterdam, The Netherlands; [5]Department of Internal Medicine, University Hospital Utrecht, The Netherlands; [6]Department of Clinical Oncology, University Hospital Leiden, The Netherlands

**Summary** In patients with metastatic renal cell carcinoma response rates of 7–26% have been achieved with immunotherapy. A high response rate of 48% in 35 patients has been reported for treatment with the combination of interferon-α (IFN-α), interleukin-2 (IL-2) and 5-fluorouracil (5-FU) (Atzpodien et al (1993*a*) *Eur J Cancer* **29A**: S6–8). We conducted a multicentre phase II study to confirm these results. Metastatic renal cell carcinoma patients were treated as outpatients with an 8-week treatment cycle. Recombinant human IL-2 20 MU $m^{-2}$ was administered subcutaneously (s.c.) three times a week (t.i.w) in weeks 1 and 4 and 5 MU $m^{-2}$ t.i.w. in weeks 2 and 3. Recombinant human IFN-α 2a 6 MU $m^{-2}$ was administered s.c. once in weeks 1 and 4 and t.i.w. in weeks 2 and 3, and 9 MU $m^{-2}$ t.i.w. in weeks 5–8. 5-FU (750 mg $m^{-2}$) was given as a bolus injection intravenous once a week in weeks 5–8. The treatment cycle was repeated once in case of response or minor response. Fifty-two patients entered the study. All had undergone a nephrectomy and had progressive metastatic disease. The median WHO-performance status was 1, the median number of metastatic sites was 2 (range 1–5) and the median time between the diagnosis of the primary tumour and the start of treatment was 12.9 months (range 1–153). Among the 51 patients, including four patients with early progressive disease, who were evaluable for response, the response rate was 11.8% (95% confidence interval (CI) 2.9–20.7%), with no complete responses. Median duration of response was 8.3 (range 3.8–22.4+) months. Median survival was 16.5 (range 1.8–30.5+) months. Grade 3/4 toxicity (WHO) occurred in 29/52 (55.8%) of the patients in cycle 1 and in 6/16 (37.5%) of the patients in cycle 2. It consisted mainly of anorexia, fatigue, nausea, fever and leucocytopenia. We cannot confirm the high response rate in patients with metastatic renal cell carcinoma treated with the combination of IFN-α, IL-2 and 5-FU, as described by Atzpodien et al. © 2000 Cancer Research Campaign

**Keywords**: immunochemotherapy; renal cell carcinoma; phase II; interleukin-2; interferon-α; 5-FU

Metastatic renal cell carcinoma has a poor prognosis. One-third of the patients with renal cell carcinoma present with metastatic disease and one-half of the other patients will develop metastases during further follow-up. The median survival is only 6 months and the 5-year-survival is less than 5% (Linehan et al, 1997).

Conventional systemic therapies such as chemotherapy and hormonal therapy generally induce response rates below 10% and have no impact on survival (Fossa et al, 1994; Kjaer, 1988). In view of the wide variability in the natural history of the disease with, on the one hand, sometimes rapid progression and, on the other hand, the occurrence of spontaneous regression of metastases, it is thought that immune mechanisms may play a role. Therefore, biologic response modifiers have undergone extensive evaluation. Interferon-α (IFN-α) and interleukin-2 (IL-2), both as single-agent and in various combinations, are the best investigated agents. IFN-α results in response rates of 8–26%, with a median survival of 13 months (Negrier et al, 1998; Creagan et al, 1991; Umeda and Niijima, 1986). IL-2 induces a response rate of 7–23%, with a median survival of 12 months (Negrier et al, 1998; Fyfe et al, 1996; Gore et al, 1994). The combination of the two cytokines has only given a slight increase in response rate (Negrier et al, 1998; Palmer et al, 1993; Atzpodien et al, 1995; Facendola et al, 1995). In randomized studies comparing the combination of IFN-α and IL-2 with each single agent no survival advantage was found (Negrier et al, 1998; Jayson et al, 1998).

To improve these results, several groups have investigated the combination of these cytokines with chemotherapy. In 1993, Atzpodien et al reported a 48% response rate (95% confidence interval (CI) 32–66%) in 35 patients with the combination of IFN-α, IL-2 and 5-fluorouracil (5-FU) (Atzpodien et al, 1993*a*). Given these promising results, we performed a multicentre confirmatory study with the same schedule in patients with metastatic renal cell carcinoma.

### PATIENTS AND METHODS

### Patients

The study was performed by the Dutch Immunotherapy Working Party and accrual took place between 1994 and 1996. All patients had histologically proven renal cell carcinoma, had undergone a nephrectomy for renal cell carcinoma and documented progression

*Received 8 March 1999*
*Revised 15 October 1999*
*Accepted 21 October 1999*

*Correspondence to:* CML van Herpen of metastases prior to entry. The following eligibility criteria for the trial applied: bidimensionally measurable disease, WHO performance status 0–1, age 18–75 years, serum values of creatinine ≤ 150 μM, bilirubin ≤ 25 μM, white cell count ≥ $4 \times 10^9$ $l^{-1}$ and platelets ≥ $100 \times 10^9$ $l^{-1}$. Previous hormonal treatment was allowed provided that the treatment was stopped for at least 2 weeks. Patients with unstable angina pectoris, recent myocardial infarction (in the last 6 months) or arrhythmia's requiring therapy, active infections, a history of second malignancy with the exception of adequately treated carcinoma in situ of the cervix or basal cell carcinoma of the skin, concurrent treatment with immunosuppressive agents, bone metastases as only metastatic site, clinical signs of central nervous system involvement, previous immuno- or chemotherapy and pregnant or lactating women were excluded. Written informed consent was obtained from all patients. Before initiation of this trial, institutional review board approval was obtained at each of the participating centres.

### Study design

The study was an open label phase II study. Patients were treated as outpatients with an 8-week treatment cycle. Recombinant human IL-2 (Chiron) 20 MU $m^{-2}$ was administered subcutaneously (s.c.) 3 times a week (t.i.w.) in weeks 1 and 4, and 5 MU $m^{-2}$ t.i.w. in weeks 2 and 3. Recombinant human IFN-α 2a (Roche) 6 MU $m^{-2}$ was administered s.c. once in weeks 1 and 4 as well as t.i.w. in weeks 2 and 3, and 9 MU $m^{-2}$ t.i.w. in weeks 5–8. 5-FU (750 mg $m^{-2}$) was given as a bolus injection intravenous (i.v.) once a week in weeks 5–8. Before and after IFN-α or IL-2 administration, patients received acetaminophen 1000 mg orally. The addition of naproxen 250 mg for constitutional symptoms caused by IFN-α and/or IL-2 was allowed. Patients were evaluated weekly for toxicity. After one 8-week treatment cycle patients were evaluated for response. CTC criteria for toxicity and WHO criteria for response were used. The treatment cycle was repeated once in case of response. In case of stable disease the treatment cycle was only repeated in case of acceptable toxicity and signs of minor response. In case of grade 3 or 4 toxicity (constitutional symptoms) the dose of IFN-α and IL-2 was reduced to 50%. The IL-2 was withdrawn in case of a serum creatinine or bilirubin that failed to return to grade I toxicity or better, or in case of myocardial ischaemia. Criteria for removal from the study were tumour progression whilst on therapy, unacceptable toxicity or intercurrent illness, preventing further treatment or patient refusal.

### RESULTS

Six centres included 52 patients. Patients' characteristics are listed in Table 1. All patients had undergone a nephrectomy and had progressive metastatic renal cell carcinoma. The median age was 57 years (range 27–72). The median WHO-performance status was 1 (0–1), the median number of metastatic sites was 2 (1–5) and the median time between the diagnosis and the start of treatment was 12.9 months (1–153). Two patients were included although they had a WHO PS of 2 (Karnofsky score of 70%). Nine patients received radiotherapy and three patients underwent a metastasectomy in the period prior to entry of the study. When the patients were separated in prognostic subgroups according to the model of Jones, approximately half of the patients belonged to the good prognostic group (Jones et al, 1993).

**Table 1** Patients' characteristics

| Characteristics | No. of patients |
|---|---|
| Total number of patients (men/women) | 52 (39/13) |
| Median age, years (range) | 57 (27–72) |
| WHO-performance status | |
| 0 | 30 |
| 1 | 20 |
| 2 | 2 |
| No. of metastatic sites | |
| 1 | 20 |
| 2 | 19 |
| 3 | 11 |
| 4 | 1 |
| 5 | 1 |
| Metastatic sites | |
| Lymph nodes | 22 |
| Lung | 42 |
| Adrenal | 3 |
| Liver | 9 |
| Bone | 6 |
| Renal | 7 |
| Pleura | 4 |
| Retroperitoneal lesion | 4 |
| Other | 3 |
| Time between diagnosis and treatment for metastases | |
| ≤ 24 months | 35 |
| > 24 months | 17 |
| Median (range) | 12.9 (1–153) |
| Prognostic subgroup[a] | |
| Good | 25 (48%) |
| Intermediate | 17 (33%) |
| Poor | 10 (19%) |

[a]Division in prognostic subgroups according to Jones et al (1993).

### Response and survival

In five patients treatment was stopped within 3 weeks after starting. One patients with rapidly progressive symptomatic bone metastasis in the second week of treatment was offered radiotherapy and systemic treatment was discontinued. In one patient in-growth of a metastasis of the pancreas in the duodenum resulted in a duodenal bleeding after 2 weeks of treatment; treatment was stopped and radiotherapy was given. Two patients showed significant early progression of their disease within 3 weeks and treatment was stopped. One patient refused further treatment after 2 weeks; he had no grade III or IV toxicity, but suffered from headaches and nausea grade II.

The patients with early progression ($n = 4$) are included in the response analysis. Thus, 51 patients were evaluable for response. After one treatment cycle, five patients (9.8%) had a partial response and there were no complete responses; 31 patients (60.8%) had stable disease and 15 (29.4%) had progressive disease.

Sixteen patients received a second cycle: four of the five patients with a partial response and 12 of the 31 patients with stable disease, but with minor signs of response. One of the five patients with a partial response did not receive the second cycle due to toxicity. After the second cycle, one patient with initially a stable disease reached a partial response. Thus, the overall response rate was 11.8% (95% CI 2.9–20.7%) in the 51 evaluable patients and 12.8% (95% CI 3.2–22.4%) in the 47 fully evaluable patients. Responses occurred most frequently in lung, but also in lymph nodes and adrenal metastases. Median response duration

© 2000 Cancer Research Campaign 

**Table 2** Grade 3/4 toxicity in 52 evaluable patients in cycle 1 and in 16 patients in cycle 2

| Toxicity | Grade 3 | | Grade 4 | | Total no. of patients (%) | |
|---|---|---|---|---|---|---|
| | Cycle 1 | Cycle 2 | Cycle 1 | Cycle 2 | Cycle 1 | Cycle 2 |
| Anorexia | 12 | 3 | 0 | 0 | 12 (23) | 3 (19) |
| Leucocytopenia | 9 | 3 | 2 | 0 | 11 (21) | 3 (19) |
| Malaise | 8 | 2 | 1 | 1 | 9 (17) | 3 (19) |
| Nausea/vomiting | 7 | 2 | 1 | 0 | 8 (15) | 2 (13) |
| Flu-like symptoms | 4 | 1 | 0 | 0 | 4 (8) | 1 (6) |
| Hypotension | 4 | 0 | 0 | 0 | 4 (8) | 0 |
| Hypertension | 1 | 1 | 0 | 0 | 1 (2) | 1 (6) |
| Diarrhoea | 1 | 0 | 0 | 0 | 1 (2) | 0 |
| Respiratory distress | 1 | 0 | 0 | 1 | 1 (2) | 1 (6) |
| Hyperbilirubinaemia | 0 | 1 | 1 | 0 | 1 (2) | 1 (6) |
| Cutaneous | 1 | 0 | 0 | 0 | 1 (2) | 0 |

Toxicity according to CTC criteria: 1, mild; 2, moderate; 3, severe; 4, life-threatening. Numbers are patients.

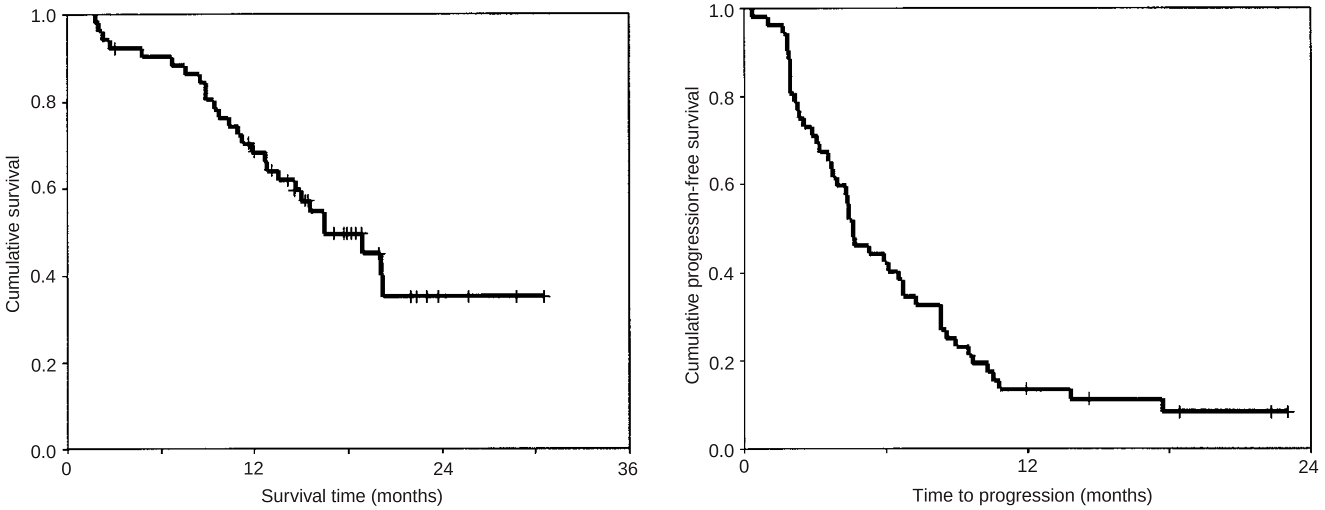

**Figure 1** (**A**) Survival from time of start of treatment. (**B**) Time to progression from time of start of treatment.

was 8.3 months (3.8–22.4+ months). Median survival was 16.5 months (1.8–30.5+ months) (Figure 1).

### Toxicity of treatment

Fifty-two patients were evaluable for toxicity. Grade 3/4 toxicity occurred in 29/52 (55.8%) of the patients in cycle 1 and in 6/16 (37.5%) of the patients in cycle 2. It consisted mainly of anorexia, fatigue, nausea, fever and leucocytopenia (Table 2).

Of the 47 fully evaluable patients, only 30 (63.8%) received a complete first cycle without reduction or delay of treatment. Nine patients (19.2%) completed the first cycle with a dose reduction or delay. Eight patients (17%) did not complete the first cycle. Of the 16 patients treated with a second cycle, 13 (81%) received a complete cycle without reduction or delay of treatment. Two patients (13%) completed the second cycle with a dose reduction or delay. Only one patient (6%) did not complete the second cycle.

### DISCUSSION

Since the 1980s a large number of studies have been performed treating patients with metastatic renal cell carcinoma with IFN-α and/or IL-2. In phase II studies these drugs are associated both as single agent and in combination, with a response rate between 6 and 25% and a median survival of 8–14 months (Umeda and Niijima, 1986; Creagan et al, 1991; Gore et al, 1994; Fyfe et al, 1996). In a randomized phase III study the response rates of patients treated with IL-2, IFN-α or the combination were 6.5%, 7.5% and 18.6% respectively. However, the overall survival rates in the three groups were not significantly different from one another, and the median survival times were 12, 13 and 17 months respectively (Negrier et al, 1998). The rationale for combining IFN-α and IL-2 is that in vitro IFN-α enhances the cell membrane expression of major histocompatibility complex (MHC) antigens to which IL-2-activated T-cells can respond (Guadagni et al, 1989).

To improve these moderate results, several groups have proposed combination of these cytokines with chemotherapy. The mechanisms underlying the supposed synergistic interaction between immunotherapy and chemotherapy are still speculative. Arguments for the enhancement of the anti-tumour activity of immunotherapy by chemotherapy as well as vice versa have been postulated. When 5-FU is used as monotherapy in patients with metastatic renal cell carcinoma, the tumour activity is weak, with a

 *© 2000 Cancer Research Campaign*

**Table 3** Phase II studies in MRCC with the combination IL-2, IFN-α, and 5-FU

| Author | Response rate (%) | Country |
|---|---|---|
| Atzpodien, 1993*b* | 48 | Germany |
| Hofmockel, 1996 | 38 | Germany |
| Joffe, 1996 | 16 | UK |
| Dutcher, 1996 | 19 | USA |
| Lopez-Hanninen, 1996 | 39 | Germany |
| Atzpodien, 1997 | 39 | Germany |
| Kirchner, 1998 | 33 | Germany |
| van Herpen, 1999 | 12 | The Netherlands |
| Sella, 1994 | 39 | USA |
| Ellerhorst, 1997 | 31 | USA |
| Negrier, 1997 | 8 | France |
| Tourani, 1998 | 20 | France |
| Ravaud, 1998 | 2 | France |
| Ventriglia, 1998 | 35 | Argentina |

Above the double line the studies that used exactly the same schedule as Atzpodien are summarized. Below that line the studies with another schedule are enumerated.

response rate less than 10% (Kish et al, 1994). However, it has been observed that the tumour cells of renal cancer acquire an increased susceptibility to lymphocyte activated killer (LAK) cells after incubation with 5-FU (Reiter et al, 1992; Tomita et al, 1993). Also, a modification in the metabolism of 5-FU by IFN-α has been reported (Pfeffer and Tamm, 1984; Seymour et al, 1994).

In 1993, Atzpodien et al used a sequential combination of IFN-α s.c., IL-2 s.c. and 5-FU i.v. and observed an objective response rate of 48% in 35 patients, with 11% complete responses (Atzpodien et al, 1993*b*). Disappointingly, in our study, in which we treated the patients with the same schedule, the response rate was only 11.8% with no complete responses. Three other groups also have used the same schedule as Atzpodien et al and found a response rate of 38, 16 and 19%, with 9, 0 and 0% complete responses respectively (Hofmockel et al, 1996; Joffe et al, 1996; Dutcher et al, 1996). The group of Atzpodien et al has extended their experience with this schedule and reported response rates of 39% in 120 patients, 39% in 41 patients and 33% in 246 patients (Lopez Hanninen et al, 1996; Atzpodien et al, 1997; Kirchner et al, 1998). The combination of IFN-α, IL-2 and 5-FU is also used in other schedules. The most important differences are that either IL-2 was given as a continuous i.v. infusion and/or the drugs were administered all three concomitantly. The response rates were 39, 31, 8, 20, 2 and 35% respectively for the reports by Sella et al (1994), Ellerhorst et al (1997), Negrier et al (1997), Tourani et al (1998), Ravaud et al (1998) and Ventriglia et al, (1998) (Table 3).

The discrepancy in results between our study and some of the other studies using the same schedule might be explained by three factors. First, patient selection based on known and unknown factors can be an important cause. Half of our patients belonged to the good prognostic subgroup according to the criteria of Jones et al (1993). Five of the six responders belonged to that group and the other to the intermediate prognostic subgroup. There was no response in the poor prognostic subgroup. It is difficult to compare our group of patients with that of Atzpodien et al, due to the use of a different prognostic system (Atzpodien et al, 1993*b*). When compared with others, our group showed more favourable prognostic factors than the patients reported by Joffe, but was similar to the group reported by Tourani (Joffe et al, 1996; Tourani et al, 1998).

A second factor that may have influenced the results is that, after nephrectomy, we waited until evidence of progression of disease was established before starting treatment. The spontaneous regression rate can be as high as 7% (Gleave et al, 1998). It is not clear if in other studies patients were also included immediately after nephrectomy.

The third reason can be the high rate of grade 3 or 4 toxicity (55.8% of the patients in the first cycle). As a consequence only 63% of the patients got the first cycle without delay or dose reduction. The grade 3 and 4 toxicity was much higher than reported in most of the other studies (9–18%) using exactly the same schedule (Hofmockel et al, 1996; Lopez Hanninen et al, 1996) but was comparable with the toxicity (44%) found by Joffe et al (1996).

In conclusion, we cannot confirm the high response rate in patients with metastatic renal cell carcinoma treated with the combination of IFN-α, IL-2 and 5-FU, described by Atzpodien et al (1993*a*). To eliminate the influence of unknown differences in the various populations a phase III study is warranted to determine the true efficacy of this schedule.

### REFERENCES

Atzpodien J, Kirchner H, Hanninen EL, Deckert M, Fenner M and Poliwoda H (1993*a*) Interleukin-2 in combination with interferon-alpha and 5-fluorouracil for metastatic renal cell cancer. *Eur J Cancer* **29A**: S6–8

Atzpodien J, Kirchner H, Hanninen EL, Korfer A, Fenner M, Menzel T, Deckert M, Franzke A, Jonas U and Poliwoda H (1993*b*) European studies of interleukin-2 in metastatic renal cell carcinoma. *Semin Oncol* **20**: 22–26

Atzpodien J, Lopez Hanninen E, Kirchner H, Bodenstein H, Pfreundschuh M, Rebmann U, Metzner B, Illiger HJ, Jakse G, Niesel T, Scholz H, Wilhelm S, Pielmeier T, Zakrzewski G, Blum G, Beier J, Müller G, Duensing S, Allhoff E, Jonas U and Poliwoda H (1995) Multiinstitutional home-therapy trial of recombinant human interleukin-2 and interferon α-2 in progressive metastatic renal cell carcinoma. *J Clin Oncol* **13**: 497–501

Atzpodien J, Kirchner H, Franzke A, Wandert T, Probst M, Buer J, Duensing S and Ganser A (1997) Results of a randomized clinical trial comparing s.c. interleukin-2, s.c. α-2a-interferon, and i.v. bolus 5-fluorouracil against oral tamoxifen in progressive metastatic renal cell carcinoma patients. *ASCO Proc* **16**: 326a

Creagan ET, Twito DI, Johansson SL, Schaid DJ, Johnson PS, Flaum MA, Buroker TR, Geeraerts LH, Veeder MH, Gesme DH, Jr and Homburger H (1991) A randomized prospective assessment of recombinant leukocyte A human interferon with or without aspirin in advanced renal adenocarcinoma. *J Clin Oncol* **9**: 2104–2109

Dutcher JP, Logan T, Gordon M, Sosman J, Weiss G, Margolin K, Plasse T and Atkins M (1996) 5-FU and subcutaneous IL-2 plus s.c. intron in metastatic renal cell cancer (RCC) patients. *ASCO Proc* **15**: 272

Ellerhorst JA, Sella A, Amato RJ, Tu SM, Millikan RE, Finn LD, Banks M and Logothetis CJ (1997) Phase II trial of 5-fluorouracil, interferon-α and continuous infusion interleukin-2 for patients with metastatic renal cell carcinoma. *Cancer* **80**: 2128–2132

Facendola G, Locatelli MC, Pizzocaro G, Piva L, Pegoraro C, Pallavicini EB, Signaroldi A, Meregalli M, Lombardi F, Beretta GD, Scanzi R, Labianca R and Luporini G (1995) Subcutaneous administration of interleukin 2 and interferon-α-2b in advanced renal cell carcinoma: a confirmatory study. *Br J Cancer* **72**: 1531–1535

Fossa SD, Kramar A and Droz JP (1994) Prognostic factors and survival in patients with metastatic renal cell carcinoma treated with chemotherapy or interferon-α. *Eur J Cancer* **30A**: 1310–1314

Fyfe GA, Fisher RI, Rosenberg SA, Sznol M, Parkinson DR and Louie AC (1996) Long-term response data for 255 patients with metastatic renal cell carcinoma treated with high-dose recombinant interleukin-2 therapy [letter]. *J Clin Oncol* **14**: 2410–2411

Gleave M, Elhilali M, Fradet Y, Davis I, Venner P, Saad F, Klotz L, Moore M, Paton V, Bajamonde A and the Canadian Urologic Oncology Group (1998) Interferon γ-1b compared with placebo in metastatic renal-cell carcinoma. *N Engl J Med* **338**: 1265–1271

*© 2000 Cancer Research Campaign*

Gore ME, Galligioni E, Keen CW, Sorio R, Loriaux EM, Grobben HC and Franks CR (1994) The treatment of metastatic renal cell carcinoma by continuous intravenous infusion of recombinant interleukin-2. *Eur J Cancer* **30A**: 329–333
Guadagni F, Schlom J, Johnston WW, Szpak CA, Goldstein D, Smalley R, Simpson JF, Borden EC, Pestka S and Greiner JW (1989) Selective interferon-induced enhancement of tumor-associated antigens on a spectrum of freshly isolated human adenocarcinoma cells. *J Natl Cancer Inst* **81**: 502–512
Hofmockel G, Langer W, Theiss M, Gruss A and Frohmuller HG (1996) Immunochemotherapy for metastatic renal cell carcinoma using a regimen of interleukin-2, interferon-α and 5-fluorouracil. *J Urol* **156**: 18–21
Jayson GC, Middleton M, Lee SM, Ashcroft L and Thatcher N (1998) A randomized phase II trial of interleukin 2 and interleukin 2-interferon α in advanced renal cancer. *Br J Cancer* **78**: 366–369
Joffe JK, Banks RE, Forbes MA, Hallam S, Jenkins A, Patel PM, Hall GD, Velikova G, Adams J, Crossley A, Johnson PW, Whicher JT and Selby PJ (1996) A phase II study of interferon-α, interleukin-2 and 5-fluorouracil in advanced renal carcinoma: clinical data and laboratory evidence of protease activation. *Br J Urol* **77**: 638–649
Jones M, Philip T, Palmer P, Von der Maase H, Vinke J, Elson P, Franks CR and Selby . (1993) The impact of interleukin-2 on survival in renal cancer: a multivariate analysis. *Cancer Biother* **8**: 275–288
Kirchner H, Buer J, Probst-Kepper M, Franzke A, Hoffmann R, Wittke F, Ganser A and Atzpodien J (1998) Risk and long-term outcome in metastatic renal cell carcinoma patients receiving s.c. interleukin-2, s.c. interferon-α2a and i.v. 5-fluorouracil. *ASCO Proc* **17**: 310a
Kish JA, Wolf M, Crawford ED, Leimert JT, Bueschen A, Neefe JR and Flanigan RC (1994) Evaluation of low dose continuous infusion 5-fluorouracil in patients with advanced and recurrent renal cell carcinoma. A Southwest Oncology Group Study. *Cancer* **74**: 916–919
Kjaer M (1988) The role of medroxyprogesterone acetate (MPA) in the treatment of renal adenocarcinoma. *Cancer Treat Rev* **15**: 195–209
Lopez Hanninen E, Kirchner H and Atzpodien J (1996) Interleukin-2 based home therapy of metastatic renal cell carcinoma: risks and benefits in 215 consecutive single institution patients. *J Urol* **155**: 19–25
Linehan MW, Shipley WU and Parkinson DR (1997) Cancer of the kidney and ureter. In: Cancer. Principles and Practice of Oncology, De Vita VT Jr, Hellman S and Rosenberg SA (eds), pp. 1271–1299. Lippincott-Raven: Philadelphia.
Negrier S, Escudier B, Douillard JY, Lesimple T, Rossi JF, Viens P, DiStefano-Louineau D, Drevon M, Gomez F, Caty A and The French Immunotherapy Group (1997) Randomized study of interleukin-2 (IL2) and interferon (IFN) with or without 5-FU (FUCY study) in metastatic renal cell carcinoma (MRCC). *ASCO Proc* **16**: 326a
Negrier S, Escudier B, Lasset C, Douillard JY, Savary J, Chevreau C, Ravaud A, Mercatello A, Peny J, Mousssseau M, Philip T and Tursz T (1998) Recombinant human interleukin-2, recombinant human interferon-α-2a, or both in metastatic renal-cell carcinoma. *N Engl J Med* **338**: 1272–1278
Palmer PA, Atzpodien J, Philip T, Negrier S, Kirchner H, Von der Maase H, Geertsen P, Evers P, Loriaux E, Oskam R, Roest G, Vinke J and Franks C (1993) A comparison of 2 modes of administration of recombinant interleukin-2: continuous intravenous infusion alone versus subcutaneous administration plus interferon α in patients with advanced renal cell carcinoma. *Cancer Biother* **8**: 123–136
Pfeffer LM, Tamm I (1984) Interferon inhibition of thymidine incorporation into DNA through effects on thymidine transport and uptake. *J Cell Physiol* **121**: 431–436
Ravaud A, Audhuy B, Gomez F, Escudier B, Lesimple T, Chevreau C, Douillard JY, Caty A, Geoffrois L, Ferrero JM, Linassier C, Drevon M and Negrier S (1998) Subcutaneous interleukin-2, interferon α-2a, and continuous infusion of fluorouracil in metastatic renal cell carcinoma: a multicenter phase II trial. Groupe Francais d'Immunotherapie. *J Clin Oncol* **16**: 2728–2732
Reiter Z, Ozes ON, Blatt LM and Taylor MW (1992) A dual anti-tumor effect of a combination of interferon-α or interleukin-2 and 5-fluorouracil on natural killer (NK) cell-mediated cytotoxicity. *Clin Immunol Immunopathol* **62**: 103–111
Sella A, Zukiwski AA, Robinson E, Kilbourn R, Ellerhorst J, Amato R and Logothetis C (1994) Interleukin-2 (IL-2) with Interferon-α (IFN-α) and 5-Fluorouracil (5-FU) in patients with metastatic renal cell cancer. *ASCO Proc* **13**: 237
Seymour MT, Patel N, Johnston A, Joel SP and Slevin ML (1994) Lack of effect of interferon α 2a upon fluorouracil pharmacokinetics. *Br J Cancer* **70**: 724–728
Tomita Y, Imai T, Katagiri A, Kimura M, Saitoh K and Sato S (1993) 5-Fluorouracil increases susceptibility of renal cell cancer cell lines to lymphokine-activated killer cells: evidence for alteration not at the level of recognition but at a post-binding stage of the lytic cycle. *Cancer Lett* **75**: 27–34
Tourani JM, Pfister C, Berdah JF, Benhammouda A, Salze P, Monnier A, Paule B, Guillet P, Chretien Y, Brewer Y, Di Palma M, Untereiner M, Malaurie E, Tadrist Z, Pavlovitch JM, Hauteville D, Mejean A, Azagury M, Mayeur D, Lucas V, Krakowski I, Larregain Fournier D, Abourachid H, Andrieu JM and Chastang C (1998) Outpatient treatment with subcutaneous interleukin-2 and interferon α administration in combination with fluorouracil in patients with metastatic renal cell carcinoma: results of a sequential non-randomized phase II study. Subcutaneous Administration Proleukin Program Cooperative Group. *J Clin Oncol* **16**: 2505–2513
Umeda T, Niijima T (1986) Phase II study of interferon α on renal cell carcinoma. Summary of three collaborative trials. *Cancer* **58**: 1231–1235
Ventriglia M, Estevez R and Tiscomia A (1998) Chemoimmunotherapy with interleukin-2, interferon-α-2b and 5-fluorouracil in outpatients with advanced renal cell carcinoma. *ASCO Proc* **17**: 347a

 *© 2000 Cancer Research Campaign*
